# Relationship between neighbourhood cohesion and disability: findings from SWADES population-based survey, Kerala, India

**DOI:** 10.12688/f1000research.25073.1

**Published:** 2020-07-13

**Authors:** M.D. Saju, Anuja Maria Benny, Komal Preet Allagh, Binoy Joseph, Jotheeswaran Amuthavalli Thiyagarajan

**Affiliations:** 1 Rajagiri College of Social Sciences (Autonomous), Cochin, Kerala, 683104, India; 2 Rajagiri International Centre for Consortium Research in Social Care (ICRS), Rajagiri College of Social Sciences, Cochin, Kerala, 683104, India; 3 Institute of Psychiatry, Psychology & Neuroscience, King's College, London, UK, SE5 9NU, UK; 4 Department of Maternal, New-born, child, adolescent health and aging, World Health Organization, Avenue Appia 20, Geneva, CH-1211, Switzerland

**Keywords:** Neighbourhood, Social cohesion, Disability, Population study, India

## Abstract

**Background: **The burden of disability on individuals and society is enormous in India, and informal care systems try to reduce this burden. This study investigated the association between neighbourhood cohesion and disability in a community-based population in Kerala, India. To the best of our knowledge, no previous studies have examined this association in India.

**Methods: **A cross-sectional household survey was conducted with 997 participants aged 30 years and above, in Kerala. Neighbourhood cohesion was assessed by three scales: trust, community participation, and perceived safety. Functional ability was measured by WHODAS 2.0. Explanatory covariates included chronic disease conditions, age, gender, education, income, and mental health conditions.

**Results:** Of 997 participants (37% male; mean age, 53.9 [range, 30–90] years), the majority were married or cohabiting. Univariate analysis showed functional ability to be positively associated with most demographic and health characteristics. However, after adjustment, only social cohesion, age, income, education, chronic diseases and mental health conditions remained significant. Mediation analysis showed the effect of personal and health characteristics on functional ability as mediated by social cohesion.

**Conclusion:** Social cohesion is an important moderator of functional ability. Interventions targeting the creation of stronger ties among neighbours and a sense of belonging should be scaled-up and evaluated in future research.

## Introduction

Of the one billion people living with some form of disability in the world, nearly 200 million experience considerable difficulties in physical functioning ( WHO, 2011). The International Classification of Functioning, Disability and Health defines disability as an umbrella term for impairments, activity limitations and participation restrictions ( WHO, 2002). Disability appears to be a biological and social phenomenon, but the person with impairment faces multifaceted issues in various domains of life, that prevent them from full participation in society ( WHO, 2011). Social exclusion prevents people with disability accessing various formal and informal services, such as health care, education, employment, social services, housing and transport ( WHO, 2015). Disability is a development issue because persons with disabilities experience increased poverty and deprivation due to the above mentioned barriers compared with persons without disabilities ( WHO, 2011). Poverty and deprivation is a predictor of increased impairments through malnutrition, poor access to health, and deprived living, working and travelling conditions. Disability creates a vicious cycle of poverty through lack of access to education and employment, and through increased out of pocket expenditure (OOP). Even a small OOP expenditure on health can put them at risk of drifting to below poverty line, especially for households that are just above the poverty line ( van Doorslaer *et al.*, 2006). Though the life expectancy of the general population has increased due to education, per capita income, living conditions and medical practices ( Wang *et al.*, 2017), life expectancy has not increased in the same extent for people with disabilities ( Murray *et al.*, 2015). Therefore, reducing morbidity is paramount for improving wellbeing of the population, as well as attaining the United Nations Sustainable Development Goals.

Parallel to epidemiological transitions, India, particularly the state of Kerala, is experiencing social, cultural and economic transitions. The key factors driving these include changes in family structures, urbanization and industrialization. These unprecedented changes influence neighbourhood cohesion, social interactions and support systems of the entire community. Several earlier studies have found a positive impact of neighbourhood social cohesion on disability ( Avlund *et al.*, 2004; Fiddian-Qasmiyeh *et al.*, 2014). Social cohesion is defined as a cohesive society that works towards the well-being of all its members, fights exclusion and marginalization, creates a sense of belonging, promotes trust, and offers its members opportunity of upward mobility ( Laiglesia, 2012).

A putative explanation for neighbourhood is often referred to as a person in a situation that includes availability of all to facilities and provisions including health care, formal and informal services, and safe, secure and supportive social engagement opportunities. There are a number of studies that have investigated the effect of neighbourhood social interaction on disability and found that neighbourhood social cohesion, the perceived degree of connectedness among neighbours and their willingness to intervene for the common good, is an important aspect ( Avlund *et al.*, 2004; Fiddian-Qasmiyeh *et al.*, 2014). Beyond that, the impact of neighbourhood cohesion and social interaction on disability and common mental issues like depression, anxiety and stress is less understood. In this study, we aim to study the association of a person’s disability with their social cohesion, and explore the channels through which disability affects social cohesion. We conducted a search on electronic databases such as, Medline, psychINFO and PubMed, using MeSH terms (social cohesion social support, disability, India), which yielded no results, concluding that no such studies have been done previously in India, to the best of our knowledge.

In the last three decades, India has experienced a major demographic, health, social and economic transitions and its impact in social cohesion has not been systematically looked at. In the context of paucity of evidence for social cohesion, this study investigates the relationship between social cohesion and functional ability after accounting for all known confounding factor,; and to what extent the effect of personal and health characteristics on functional ability is mediated by social cohesion.

## Methods

### Study design, setting and participants

The present study is based on data collected during the ‘Social well-being and determinants of health’ SWADES study Families of the SWADES cohort were invited to be part of the baseline questionnaire between April and May 2018 ( Saju *et al.*, 2020a). The study catchment area was located in semi-rural region of Keezhmadu panchayat in Ernakulam, Kerala, India. The residents had mixed culture and socioeconomic background. Catchment area boundaries were precisely defined. Mapping was carried out to identify and locate all households. All the family members residing in that geographically located area aged 30 years and above were considered for the study. The objectives of the SWADES study are as follows: a) to monitor changes over time in physical, behavioural and social risk factors associated with chronic diseases and mental health comorbid conditions; b) to develop chronic disease risk prediction models and estimate the probability of having or developing a particular chronic disease within a specified period; and c) to scale up population and family health interventions and evaluate the impact. SWADES collected data pertaining to sociodemographic, physical, mental, functional, social cohesion, social support networks, behavioural risk factors, health services utilisation, cognitive function and risk of fall using respective scales or tools. A detailed description of the SWADES study and the recruitment of the sample population is presented in the SWADES protocol paper ( Saju *et al.*, 2020b).

Participants provided written informed consent and the study was approved by the Institutional Review Board of Rajagiri Hospital (Study Reference Number: RAJH 18003).

### Data collection

The SWADES baseline data was used for the present study, and this study covered only two aspects of the SWADES full survey – Functional Ability and Social Cohesion.

We used a core minimum data set with cross-culturally validated assessments (mental conditions, physical health, demographics, extensive non-communicable disease risk factor questionnaires, disability/functioning and health service utilization). All study instruments were translated, back-translated, and assessed for acceptability and conceptual equivalence. Translations were done locally, by investigators fluent in English (the language of the instruments) and in the local language (Malayalam) to be used in the study. The local version was reviewed by experts in Kerala.

***Sociodemographic and health conditions.*** Education level was ascertained, and coded as: no education, did not complete primary, completed primary, completed secondary and completed tertiary education. Type of family was described as: living alone, nuclear family, extended family and mixed family. Income was a continuous variable and was coded as quartile. We also recorded age, sex, marital status (single, married/cohabitating or divorced/widowed/separated), current occupational status, and family income.

Self-reported health conditions, such as stroke, diabetes, hypertension, heart disease, were reported by participants.

***Measures of functional ability.*** The functional ability of each participant was measured by the World Health Organization Disability Assessment Schedule 2.0 (WHODAS 2.0), measuring health and disability ( Marti & Choi, 2020). The instrument covers six domains: 1) understanding and communicating with the world; 2) moving and getting around; 3) self-care; 4) getting along with people; 5) life activities; and 6) participation in society. Scores for each question range from 0 (no difficulty) to 4 (extreme difficulty/cannot do). The standardized global score ranges from 0 (non-disabled) to 100 (maximum disability) and this score was divided into quintiles. This measurement has been extensively validated in India and other low and middle-income countries ( Thomas *et al.*, 2016).

***Measures of social cohesion.*** Social cohesion was measured from the inner to the outer social circle of the participants through three items, including trust in neighbours and co-workers, community participation, perceived safety in the participant’s residential neighbourhood and general social trust. These three items were measured using a scale (see below) that has been extensively used in research conducted in low and middle income countries ( Fernández-Niño *et al.*, 2019; Kulkarni & Shinde, 2015; Ramlagan *et al.*, 2013).

Trust in neighbours and co-workers: The scale was standardized for convenience of interpretation. The subject responded to three questions about trust according to the response options ‘to a very great extent’, ‘to a great extent’, ‘neither great nor small extent’, ‘to a small extent’ and ‘to a very small extent’:

Trust in people in the participants’ neighbourhood;
Trust in people with whom the participants work; and
Trust in strangers.

Community participation: This item was measured with a standardized scale used in the Social Cohesion section of the SAGE questionnaire ( Kulkarni & Shinde, 2015), which indicates the frequency of involvement in community activities in the last 12 months. The subject responded to nine activities with the response options ‘never’, ‘once or twice per year’, ‘once or twice a month’, ‘once or twice a week’ or ‘daily’:

Attending public meetings in which there was discussion of local or school affairs;
Meeting personally with a community leader;
Attending any group, club, society, union, or organization meeting;
Working with people in the neighbourhood to fix or improve something;
Having friends over to one’s home;
Being in the home of someone who lives in a different neighbourhood;
Socializing with co-workers outside of work;
Attending religious services (excluding weddings and funerals); and
Getting out to attend social meetings, activities, programs, or events or to visit relatives or friends.

Perceived safety in the participant’s residential neighbourhood: This was assessed based on two questions. The respondent answered to each according to the response options ‘completely safe’, ‘very safe’, ‘moderately safe’, ‘slightly safe’ or ‘not safe at all’:

How safe from crime and violence the participant feels when he or she is alone at home; and
How safe the respondent feels when walking down his/her street alone after dark.

All three variables were computed separately and standardized versions of three variables, trust, safety and participation were created using ‘egen’ command in STATA. Value range was then defined from 0 to 30. Based on this score, social cohesion was divided into five quintiles with lowest value in Quintile 1 (Q1) and highest value in Quintile 5 (Q5). Respondents in Q1 has lowest Social Cohesion and respondents in Q5 has highest Social Cohesion.

### Statistical analysis

All statistical analysis was performed using STATA 14 and R version 3.6.3 We first compared the demographic characteristics of study participants using chi square tests, t-tests or Wilcoxon rank-sum tests, as appropriate to evaluate statistical significance. To evaluate the variables associated with the outcome of interest, we performed linear regression and calculated 95% confidence interval, and p-values to evaluate the statistical significance. Using R software, we plotted a jitter plot to study the association of disability with social cohesion. Finally, we performed path analysis to develop a hypothesis on probable causal relationship and recommendations for intervention development.

## Results

### Participant characteristics

We interviewed 997 participants, aged 30 years and above and residing in 573 households of the study area. The mean age of the population was 53.9 ± 14.2 years. The majority were married or cohabiting (82.7%). More than half (54%) of the population had completed primary education. Almost all (89%) the participants were born in a village. More than half of the women were housewives (50.5%) compared to 5% of men who were house husbands. Men who were employed with paid work were triple the number of women (61.4% vs. 18.5%), highlighting a patriarchal societal set-up. Two-fifths (41.8%) of the population had very low income. More than half (60.9%) of the population resided in a nuclear family (nuclear family consists of a couple and their dependent children; higher percentage of nuclear family imply lesser number of family carers for the care of people with disability, especially in the Indian context). The percentage distribution of the sample by selected socio-demographic variables and gender is presented in Table 1.

**Table 1.** Demographic characteristics of the study population.

Variable	Total, n (%)	Female, n (%)	Male, n (%)	Statistics
n	997	632	365	
**Age, years**				
30–40	203 (20.4)	61 (22.5)	142 (16.7)	chi ^2^(4) = 10.77 P= 0.029
40–50	202 (20.3)	66 (21.5)	136 (18.1)	
50–60	223 (22.4)	81 (22.5)	142 (22.2)	
60–70	228 (22.9)	96 (20.9)	132 (26.3)	
70+	141 (14.1)	61 (12.7)	80 (16.7)	
**Marital status**				
Unmarried	18 (1.8)	7 (1.1)	11 (3.0)	chi ^2^(2) = 63.13 P= 0.000
Married/cohabiting	824 (82.7)	484 (76.6)	340 (93.2)	
Widowed/ divorced/ separated	155 (15.6)	141 (22.3)	14 (3.8)	
**Education**				
No formal education	41 (4.1)	35 (5.5)	6 (1.6)	chi ^2^(3) = 10.27 P = 0.016
Primary education	538 (54.0)	333 (52.7)	205 (56.2)	
Secondary education	216 (21.)	131 (20.7)	85 (23.3)	
Above secondary	202 (20.3)	133 (21.0)	69 (18.9)	
**Place born**				
Town	77 (2.9)	58 (9.2)	19 (5.2)	chi ^2^(2) = 7.46 P = 0.024
City	29 (7.7)	22 (3.5)	7 (1.9)	
Village	891 (89.4)	552 (87.3)	339 (92.9)	
**Type of family**				
Alone	11 (1.1)	10 (1.6)	1 (0.3)	chi ^2^(3) = 8.21 P = 0.042
Nuclear Family	607 (60.9)	381(60.3)	226 (61.9)	
Extended Family	312 (31.3)	206 (32.6)	106 (29)	
Mixed Family	67 (6.7)	35 (5.5)	32 (8.8)	
**Occupation**				
Unemployed	239 (24)	170 (26.9)	69 (18.9)	chi ^2^(3) = 305.29 P = 0.000
Paid work	341 (34.2)	117 (18.5)	224 (61.4)	
Housewife/husband	337 (33.8)	319 (50.5)	18 (4.9)	
Retired	80 (8.0)	26 (4.1)	54 (14.8)	
**Income**				
Quartile 1	417 (41.8)	269 (42.6)	148 (40.6)	chi ^2^(3) = 3.59 P = 0.30
Quartile 2	108 (10.8)	62 (9.8)	46 (12.6)	
Quartile 3	245 (24.6)	163 (25.8)	82 (22.5)	
Quartile 4	227 (22.8)	138 (21.8)	89 (24.4)	
**Social cohesion**				
Quintile 1	245 (24.6)	184 (29.1)	61 (16.7)	chi ^2^(4) = 43.69 P = 0.000
Quintile 2	226 (22.7)	161(25.5)	65 (17.8)	
Quintile 3	154 (15.5)	85 (13.5)	69 (18.9)	
Quintile 4	188 (18.9)	113 (17.9)	75 (20.6)	
Quintile 5	184 (18.5)	89 (14.1)	95 (26.0)	
**Disability**				
Quintile 1	319 (32.0)	160 (25.3)	159 (43.6)	chi ^2^(4) = 49.20 P= 0.000
Quintile 2	105 (10.5)	65 (10.3)	40 (10.9)	
Quintile 3	195 (19.6)	123 (19.5)	72 (19.7)	
Quintile 4	187 (18.8)	133 (21.0)	54 (14.8)	
Quintile 5	191 (19.2)	151 (23.9)	40 (11.0)	

### Functional ability of participants

Among the study population, the prevalence of disability based on the WHO DAS score was 19.2%. Univariate analysis was employed to identify the crude association between disability and other exploratory variables ( Table 2). The prevalence of disability is twice the amount in women as compared to men (23.9% versus 11.0%). Disability prevalence increased with increase in age, lack of formal education, and presence of one or more physical or mental health condition. Participants who were widowed, divorced or separated had a higher prevalence of disability compared to married or unmarried participants. The disability scores were higher for the participants with lower economic status.

**Table 2.** Association between disability and covariates.

Explanatory variables	Crude association coefficient (95% CI)	Adjusted association coefficient (95% CI)
**Social cohesion**		
Quintile 1	1 ref	1 ref
Quintile 2	-3.7 (-5.4 to -2.0), p =0.000	-2.2 (-3.6 to -0.8), p=0.003
Quintile 3	-6.0 (-7.9 to -4.0), p =0.000	-3.3 (-5.0 to -1.7), p=0.00
Quintile 4	-6.1 (-8.0 to -4.2), p =0.000	-3.3 (-4.8 to -1.7), p =0.000
Quintile 5	-5.8 (-7.7 to -4.0), p=0.000	-2.3 (-3.9 to -0.7), p=0.005
**Age**	0.2 (0.2 to 0.3), p <0.01	0.2 (0.2 to 0.3), p <0.001
**Gender**		
Female	1 ref	1 ref
Male	-3.2 (-4.5 to -1.9), p <0.001	-1.3 (-2.6 to -0.03), p=0.044
**Marital status**		
Unmarried	1 ref	1 ref
Married/cohabiting	-1.26 (-5.6 to 3.1), p=0.57	-1.05 (-4.8 to 2.6), p=0.57
Widowed/divorced/separated	8.43 (3.9 to 13), p<0.001	1.24 (-2.8 to 5.2), p=0.54
**Education**		
No formal education	1 ref	1 ref
Primary education	-13.78 (-16.7 to -10.8), p<0.001	-8.10 (-10.7 to 5.5), p<0.001
Secondary education	-16.40 (-19.5 to-13.3), p<0.001	-7.40 (-10.2 to -4.6), p<0.001
Above secondary	-19.03 (-22.2 to-15.9), p<0.001	-8.4 (-11.3 to -5.5), p<0.001
**Place born**		
Town	1 ref	
City	2.14 (-2.1 to 6.4), p=0.325	--
Village	2.36 (-1.3 to 6.1), p=0.209	--
**Type of family**		
Alone	1 ref	
Nuclear Family	-4.16 (-10.1 to 1.8), p=0.17	--
Extended Family	-4.54 (-10.5 to 1.5), p=0.13	
Mixed Family	-1.45 (-7.8 to 4.9), p=0.65	
**Type of job**		
Unemployed	1 ref	1 ref
Paid work	-7.9 (-9.5 to -6.3), p<0.001	-2.20 (-3.7 to -0.7), p=0.004
Housewife/husband	-4.20 (-5.8 to -2.6), p=0.57	-2.60 (-4 to -1.2), p<0.001
Retired	-4.83 (-7.3 to -2.4), p=0.57	-4.24 (-6.4 to -2.1), p<0.001
**Income**		
Quartile 1	1 ref	1 ref
Quartile 2	-2.18 (-4.3 to -0.1), p=0.04	-0.98 (-2.7 to 0.7), p=0.24
Quartile 3	-0.87 (-2.4 to 0.7), p=0.27	-0.05 (-1.3 to 1.2), p=0.93
Quartile 4	-3.81(-5.4 to -2.2), p<0.001	-1.68 (-3.0 to - 0.3), p=0.014
**Physical condition**		
Absence of chronic illness	1 ref	1 ref
Presence of any one chronic illness	2.90 (1.5 to 4.3), p<0.01	0.18 (-1.0 to 1.4), p=0.76
More than one chronic illness	8.03 (6.4 to 9.6), p<0.01	1.68 (0.2 to 3.2), p=0.02
**Mental health condition (depression, anxiety)**		
No mental health condition	1 ref	1 ref
Presence of mental health condition	8.44 (7.1 to 9.8), p<0.001	6.42 (5.25 to 7.58), p<0.001

There was significant negative association between social cohesion and disability (-5.8 (-7.7 to -4), p=0.000). It was also observed that there was an independent association between people’s trust in neighbourhood (-0.57 (-0.83 to -0.30), p<0.001) and community participation (-0.38 (-0.47 to -0.29), p<0.001) with the disability.

### Social cohesion score

The mean social cohesion score was 17.04 (± 6.70). Men had a higher chance of social cohesion compared to women (1.13 (0.80 to 1.47); p<0.001). Those above the age of 70 were found to have lower social cohesion compared to the other age groups (-1.04 (-1.61 to -0.47); p<0.001). While looking into the marital status of the respondents, married respondents had a socially active life compared to unmarried, widowed or divorced (1.37 (0.14 to 2.59); p=0.029). Compared to those who had no formal education, individuals with formal education took part in more social activities (1.27 (0.44 to 2.10), p= 0.003).

### Association between disability and social cohesion

**Figure 1.**
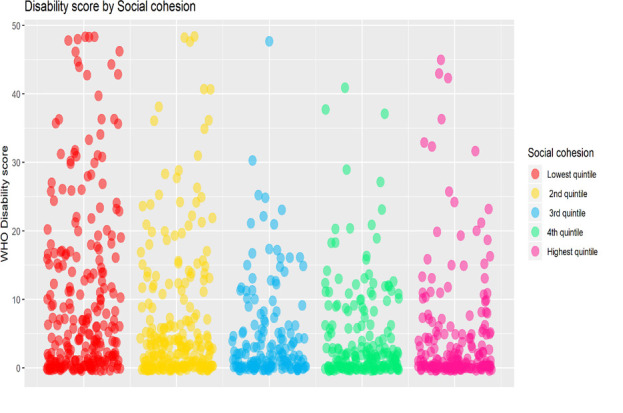
Relationship of disability score among participants residing in high and low social cohesion neighbourhoods in Kerala, India.

Figure 1 shows the association of disability with social cohesion. It shows that individuals with higher disability score reside in neighbourhoods with low social cohesion.

Multivariate analysis was performed to understand the association between social cohesion and disability after controlling for the confounding variables age, gender, education, marital status, occupation, income, physical condition and mental health condition ( Table 2). With the influence of the confounding variables, the coefficient value was reduced, but the results remained statistically significant. There was still a strong negative association between social cohesion and disability. This indicates that individuals with lower participation in the community, trust and safety exhibited higher levels of disability.

### Modelling of relationships between health and social cohesion

**Figure 2.**
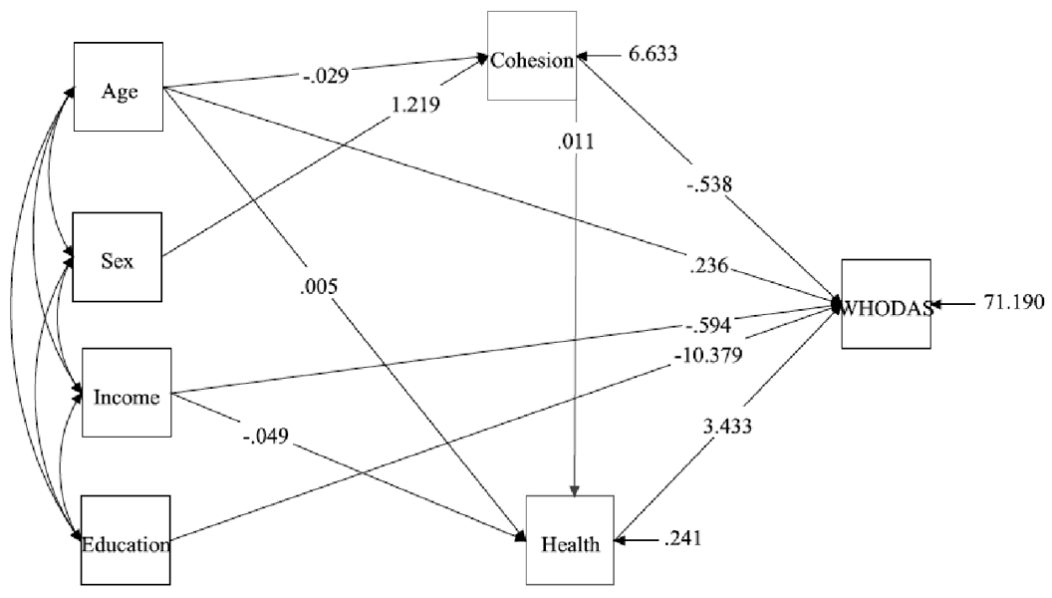
Mediation effect of social cohesion on functional ability of the population.

*Structural model:* First, we tested the direct effect of social cohesion (predictor variable) on disability (dependent variable) without mediators. The directly standardized path coefficient was not significant. Subsequently, the mediation model was tested, which included three mediators of mental health (depression, anxiety and stress) and a direct path from social cohesion to disability. The results showed that the model was a very good fit to the data. After adding the demographic variables, such as age, gender, income, education, as shown in Figure 2, the mediation model was tested again. The final meditation model showed a very good fit to the data.

Taken together, these results show the important role of social cohesion in the relationship between mental health (depression, anxiety and stress) and disability. The effect of social cohesion on disability through mental health is very high. The model revealed that demographic variables, such as, age, income, education and mental health, had a direct effect on disability. In addition, social cohesion had an indirect effect on disability through mental health.

## Discussion

To the best of our knowledge, this is the first study to examine the association between neighbourhood cohesion and disability among a community-based population in Kerala, India. In the overall sample, we found a significant association between social cohesion and disability, which suggested an increase in neighbourhood social cohesion would decrease the chance of disability. This association was pertinent even after controlling for a number of confounding variables. This study suggests that neighbourhood social cohesion may exert an effect on Indian population’s disability status.

The findings of our study are consistent with previous studies and add to the evidence. Existing literature confirms that low social cohesion increases poor self-rated health and long-term disability ( Avlund *et al.*, 2004; Hyyppä & Mäki, 2001; Laiglesia, 2012; Pampalon *et al.*, 2007). Previous studies found women to be more responsive to neighbourhood environmental influences because of lower levels of labour market participation and higher levels of family care and time spent at home, as opposed to men who may be spending more time away from the neighbourhood ( Patel *et al.*, 2012; Wen & Zhang, 2009). However, our study showed that men had a higher chance of social cohesion irrespective of most of them being employed. Similar to our findings, previous studies also describe that older adults, those without a formal education, individuals living alone or in nuclear families, and low income individuals as having lower levels of social cohesion ( Pampalon *et al.*, 2007; Rahman & Singh, 2019). Moreover, living in a neighbourhood with low social cohesion increases psychological distress ( Erdem, 2019; Rios *et al.*, 2012). A negative association between social cohesion and disability is consistent with other studies that analysed social cohesion and physical activity. In addition, area of residence and neighbourhood affect physical activity ( Vancampfort *et al.*, 2018; Yip *et al.*, 2016); higher levels of neighbourhood social cohesion, social participation and trust affects physical activity ( Legh-Jones & Moore, 2012), which is essential for reducing disability.

Neighbourhood social cohesion can be considered as a type of social support that is available in the neighbourhood social environment outside of family and friends, which effectively results in the creation and reinforcement of neighbourhood norms ( Kim *et al.*, 2014). Neighbourhood social cohesion seems to reduce stress, improve social connections and enforce norms, which ultimately supports a reduction in disability among individuals.

### Strengths and limitations

Our study had a few limitations. This is a cross-sectional study, which limits our ability to make causal inferences, like whether disability is pushing individuals to a poorer neighbourhood or whether the poorer neighbourhood is the pertinent factor for disability. Second, since the sample comprised of participants from catchment areas of one state in India, the results cannot be generalised. Third, though the social cohesion scale is extensively used in low and middle income countries ( Kulkarni & Shinde, 2015; Fernández-Niño *et al.*, 2019; Ramlagan *et al.*, 2013), it is yet to be culturally validated in our population. Third, most participants were born in villages and nearly half of the population belonged to the low income category. Though the data was taken from respondents residing in that area for more than one year, it does not allow us to adjust for the duration participants were living in their respective neighbourhoods.

Nonetheless, our study adds to the literature by presenting evidence on the association of neighbourhood social cohesion and disability conducted in low and middle income countries. Data from our study gives insight to the extent by which the effect of personal and health characteristics on functional ability is mediated by social cohesion. Moreover, data was collected only from the individuals residing in the study area for more than one year, and this indicates a defined neighbourhood.

### Implications

The study shows that there is a need to focus on neighbourhood or community level aspects along with individual level aspects to ensure increased quality of life for people with disability. Interventions targeting the entire community can bring about behaviour change in individuals, such as increased physical activity, as we can argue that those who receive positive support for physical activity tend to be more physically active. Our study also calls for a need for interventions offering community-based services, which enhance neighbourhood trust, safety and participation, need to be deployed. Government and policymakers should aim for strategies that enhance neighbourhood cohesion by integrating the physical, public and social health of its people. This will reduce accessibility, availability and affordability issues with respect to disability, as well as increase living standards of people.

## Conclusion

The present study proposes that there is a significant association between social cohesion and disability, as people with more participation in the community have increased trust and safety and are more likely not to be disabled. Results suggest that improvements in neighbourhood cohesiveness can reduce the level of disability. Future studies can focus on community level interventions that aim to socially integrate people in poorer neighbourhoods, and the instrument used in the present study can be used toassess social cohesion in future studies. A longitudinal study with a more representative sample to help understand the reasons for and strategies to overcome low neighbourhood cohesiveness among people with disability are also essential. Neighbourhood social cohesion seems to reduce stress, improve social connections and enforce norms. Further studies are however needed to study this extensively.

## Data availability

### Underlying data

Figshare: Relationship between neighbourhood cohesion and disability: findings from SWADES population-based survey, Kerala, India, https://doi.org/10.6084/m9.figshare.12610607.v3 ( Saju *et al.*, 2020c).

### Extended data

Figshare: Relationship between neighbourhood cohesion and disability: findings from SWADES population-based survey, Kerala, India, https://doi.org/10.6084/m9.figshare.12610607.v3 ( Saju *et al.*, 2020c).

This project contains the following extended data:

- Questionnaire consisting of questions used for this study (both English and local language - Malayalam)

### Reporting guidelines

STROBE checklist for ‘Relationship between neighbourhood cohesion and disability: findings from SWADES population-based survey, Kerala, India’, https://doi.org/10.6084/m9.figshare.12610607.v3 ( Saju *et al.*, 2020c).

Data are available under the terms of the Creative Commons Zero "No rights reserved" data waiver (CC0 1.0 Public domain dedication).
