# Supplementary material for: Relationship between neighbourhood cohesion and disability: findings from SWADES population-based survey, Kerala, India
Source: F1000Res. 2020 Jul 13;9:700. [Version 1] doi: 10.12688/f1000research.25073.1 (PMC7424915; doi:10.12688/f1000research.25073.1)
Supplement: Peer review history

# Peer review: Reviewer response for version 1

**DOI:** 10.5256/f1000research.27662.r68098

**Published:** 2020-08-12

**Authors:** Debbie Gioia

**Affiliations:** 1 School of Social Work, University of Maryland, Baltimore, MD, USA

Thank you for the opportunity to review this important study. I appreciate the authors concisely laid out manuscript and the rationale for the need for this work. I fully appreciate the novel nature of this study and for that reason alone, it is an important component of understanding the effects of neighborhoods on individuals and their health outcomes.

 I confess that I do not have expertise in studies involving large data sets and the types of statistical analysis required to examine the findings from the variable relationships in those data sets, as I am predominately a qualitative researcher. I hope that other reviewers will be able to speak to the methodology chosen.

 As a mental health researcher, my interest is in the types of reported mental health disorders and community responses, especially during the pandemic. I wonder if there is more specificity in the data set about types of disorders and also the comorbidity with physical health disorders.

 The source data and conclusions seem very adequate to support the article. Minor edits may still be needed but may also be a result of formatting to the journal's submission portal.

Is the work clearly and accurately presented and does it cite the current literature?

Yes

If applicable, is the statistical analysis and its interpretation appropriate?

I cannot comment. A qualified statistician is required.

Are all the source data underlying the results available to ensure full reproducibility?

Yes

Is the study design appropriate and is the work technically sound?

Yes

Are the conclusions drawn adequately supported by the results?

Yes

Are sufficient details of methods and analysis provided to allow replication by others?

Yes

Reviewer Expertise:

I am only assessing the basic topic of the research and its merits for publication especially as a novel article where no other published research exists.

I confirm that I have read this submission and believe that I have an appropriate level of expertise to confirm that it is of an acceptable scientific standard.

# Peer review: Reviewer response for version 1

**DOI:** 10.5256/f1000research.27662.r68096

**Published:** 2020-07-31

**Authors:** Jed W. Metzger

**Affiliations:** 1 Nazareth College, Rochester, NY, USA

This is an excellent and important article reflecting on a very high level professional piece of research. Overall, the one critique I have is that it is too short for the type of research - a multivariate study.

 A few specific points to make:

- The authors are encouraged to spend a little more time developing "neighborhood" as an aspect of social cohesion. For instance the males in the study have greater social cohesion- is this due to work outside the home?. For instance perhaps the females spend more of their day as home makers in the community and would have more opportunity for neighborhood social cohesion? 
- The authors found 19% of the sample had a disability- is this what the data would expect for Kerala, India?
- Clearly gender matters- more discussion on this including in promoting social cohesion with those with a disability- it would seem that efforts for males and females may be best if they are gender focused.
- Again this is a very high quality research design. A lot of the strong evidence is in figure two- please expand discussion on that figure.
- In the discussion section, please return to the neighborhood discussion.
- This paper most certainly should be accepted for indexing with these expansions

Is the work clearly and accurately presented and does it cite the current literature?

Yes

If applicable, is the statistical analysis and its interpretation appropriate?

Yes

Are all the source data underlying the results available to ensure full reproducibility?

Partly

Is the study design appropriate and is the work technically sound?

Yes

Are the conclusions drawn adequately supported by the results?

Yes

Are sufficient details of methods and analysis provided to allow replication by others?

Partly

Reviewer Expertise:

Community organizing, suicide and homicide research, international research

I confirm that I have read this submission and believe that I have an appropriate level of expertise to confirm that it is of an acceptable scientific standard.
